# Symptom Burden in Adolescents and Young Adults With Cancer

**DOI:** 10.1001/jamanetworkopen.2025.27421

**Published:** 2025-08-18

**Authors:** Michael H. Storandt, Zhaohui Jin, Kathryn J. Ruddy, Deirdre R. Pachman, Oudom Kour, Veronica Grzegorczyk, Kurt Kroenke, Joan M. Griffin, Jessica D. Austin, Sandra A. Mitchell, Ashley Wilder Smith, Shawna Ehlers, Linda L. Chlan, Ewan K. Cobran, Jacob R. Greenmyer, Wendy A. Allen-Rhoades, Qian Shi, Andrea L. Cheville

**Affiliations:** 1 Mayo Clinic, Rochester, Minnesota; 2 Indiana University School of Medicine, Indianapolis; 3 Mayo Clinic, Phoenix, Arizona; 4 National Cancer Institute, Bethesda, Maryland

## Abstract

This cross-sectional study analyzes patient-reported outcome findings from the Enhanced, Electronic Health Record–Facilitated Cancer Symptom Control (E2C2) trial to investigate level of symptom burden in adolescents and young adults with cancer.

## Introduction

In recent years, the incidence of cancer in younger people has been increasing.^1^ Adolescents and young adults (AYAs) with cancer may experience poor health-related quality of life and a high incidence of mental health disorders.^2,3^ We assessed the level of symptom burden among AYAs with cancer using patient-reported outcomes and identified factors associated with greater symptom severity among AYAs.

## Methods

This cross-sectional study worked with The Enhanced, EHR (electronic health record)-Facilitated Cancer Symptom Control (E2C2) trial, a cluster randomized pragmatic trial examining the effectiveness of routine symptom assessment and guideline-based management for patients with cancer.^4^ This trial was conducted between March 2019 and January 2023, and at enrollment, patients began reporting severity of 6 symptoms—sleep disturbance, pain, physical function impairment, anxiety, depression, and energy deficit and/or fatigue (SPPADE)—using an 11-point numerical rating scale. Symptom severity was classified as none to mild (0-3), moderate (4-6), or severe (7-10).

We completed a post hoc analysis evaluating SPPADE symptom scores reported on the first survey completed and compared demographics and symptom severity among AYAs (between ages 18-39 years at first survey) with older adults (≥40 years at first survey) using the Pearson χ^2^ test of independence. We then completed a multivariable logistic regression analysis, using backward stepwise regression to select factors for the multivariable model, for each SPPADE symptom. *P* < .008 was deemed significant using the Bonferroni correction method. Result reporting adhered to STROBE guidelines. The Mayo Clinic institutional review board approved the parent trial with a waiver of informed consent in accordance with 45 CFR §46. Data were analyzed using R version _____ (R Project for Statistical Computing). Detailed methodology is included in the eMethods in Supplement 1.

## Results

The E2C2 trial assigned surveys to 50 207 patients; 40 659 completed at least 1 SPPADE questionnaire, including 2598 AYAs. A greater portion of AYAs were female, in urban settings, single, employed, college-educated, with non-government insurance, with higher rates of sarcoma and brain cancers, and with lower incidence of metastatic disease, when compared with older adults (Table 1).

**Table 1.** Patient Demographics and Cancer Characteristics

Characteristic	Patients, No. (%)		
	Adolescents and young adults (n = 2598)	Adults ≥40 y (n = 38 061)	All (N = 40 659)
Sex			
Male	998 (38.4)	16 366 (43.0)	17 364 (42.7)
Female	1599 (61.5)	21 695 (57.0)	23 294 (57.3)
Age, median (IQR), y	34.5 (18.0-40.0)	66.1 (40.0-101.6)	65.1 (18.0-101.6)
Age group			
18-24	277 (10.7)	NA	277 (0.7)
25-31	631 (24.3)	NA	631 (1.6)
32-39	1690 (65.1)	NA	1690 (4.2)
Race			
Additional groups^a^	322 (12.4)	2005 (5.3)	2327 (5.7)
White	2276 (87.6)	36 056 (94.7)	38 332 (94.3)
Ethnicity			
Hispanic or Latino	83 (3.2)	385 (1.0)	468 (1.2)
Not Hispanic or Latino	2501 (96.3)	37 453 (98.4)	39 954 (98.3)
Unknown, No.	14	223	237
Population density			
Urban	1708 (65.7)	20 990 (55.1)	22 698 (55.8)
Micropolitan	346 (13.3)	6628 (17.4)	6974 (17.2)
Rural	509 (19.6)	10 207 (26.8)	10 716 (26.4)
Unknown, No.	35	236	271
Marital status			
Married	1533 (59.0)	27 323 (71.8)	28 856 (71.0)
Divorced or separated	106 (4.1)	3421 (9.0)	3527 (8.7)
Single	946 (36.4)	3640 (9.6)	4586 (11.3)
Widowed	4 (0.2)	75 (0.2)	79 (0.2)
Unknown, No.	9	3602	3611
Insurance payer			
Nongovernment	2028 (78.1)	13 930 (36.6)	15 958 (39.2)
Government	547 (21.1)	23 919 (62.8)	24 466 (60.2)
Unknown, No.	23	212	235
Employment			
Employed	1886 (72.6)	14 242 (37.4)	16 128 (39.7)
Disabled	98 (3.8)	1493 (3.9)	1591 (3.9)
Not employed, student, or military	591 (22.7)	2675 (7.0)	3266 (8.0)
Retired	7 (0.3)	19 534 (51.3)	19 541 (48.1)
Unknown, No.	16	117	133
Education			
Master’s or doctoral degree	339 (13.0)	4648 (12.2)	4987 (12.3)
Bachelor’s degree	643 (24.8)	6129 (16.1)	6772 (16.7)
Some college or associate’s degree	569 (21.9)	8721 (22.9)	9290 (22.8)
High school or less	323 (12.4)	8492 (22.3)	8815 (21.7)
Unknown, No.	724	10 071	10 795
Primary malignant neoplasm			
Solid malignant neoplasm	2481 (95.5)	35 438 (93.1)	37 919 (93.3)
Gastrointestinal	487 (18.7)	9269 (24.4)	9756 (24.0)
Breast	574 (22.1)	8302 (21.8)	8876 (21.8)
Lung	51 (2.0)	3910 (10.3)	3961 (9.7)
Genitourinary	176 (6.8)	3512 (9.2)	3688 (9.1)
Gynecologic	145 (5.6)	2423 (6.4)	2568 (6.3)
Head and neck	86 (3.3)	1651 (4.3)	1737 (4.3)
Sarcoma	276 (10.6)	1553 (4.1)	1829 (4.5)
Brain	392 (15.1)	1186 (3.1)	1578 (3.9)
Melanoma	119 (4.6)	1141 (3.0)	1260 (3.1)
Endocrine	104 (4.0)	1047 (2.8)	1151 (2.8)
Skin	10 (0.4)	623 (1.6)	633 (1.6)
Multiple primaries	16 (0.6)	518 (1.4)	534 (1.3)
Nervous system	28 (1.1)	101 (0.3)	129 (0.3)
Carcinoid	0 (0)	29 (0.1)	29 (0.1)
Uncertain or unspecified	17 (0.7)	173 (0.5)	190 (0.5)
Hematologic malignant neoplasm	117 (4.5)	2623 (6.9)	2740 (6.7)
Metastatic disease			
Metastatic	716 (24.2)	13 402 (35.2)	14 118 (34.7)
Not metastatic	1345 (51.8)	15 810 (41.5)	17 155 (42.2)
Uncertain, No.	537	8849	9386

Abbreviation: NA, not applicable.

^a^ Additional racial groups included African American, Asian or Pacific Islander, and other or unknown.

With regard to symptom burden, AYAs reported higher rates of moderate (26.1% vs 18.9%) and severe (10.7% vs 7.7%) anxiety (*P* < .001) and moderate (20.8% vs 17.1%) and severe (7.4% vs 6.5%) depression (*P* < .001) compared with older adults. AYAs less often reported moderate (27.9% vs 28.4%) and severe (13.3% vs 15.6%) fatigue (*P* = .002) and moderate (21.7% vs 26.7%) and severe (6.8% vs 11.1%) physical function impairment (*P* < .001). There was no difference in moderate (18.1% vs 19.2%) or severe (8.5% vs 9.0%) pain (*P* = .02) or moderate (22.2% vs 23.1%) or severe (10.4% vs 10.9%) sleep disturbance (*P* = .30) between AYAs and older adults.

In a multivariable analysis, female sex, micropolitan area code, divorced and/or separated relationship status, government insurance, being disabled, and being unemployed (including students and/or military) were associated with severe symptomology among AYAs (Table 2). Primary tumor site and metastatic disease status were not associated with severe symptomology.

**Table 2.** Multivariable Analysis of Factors Associated With Having a Severe Symptom Among Adolescents and Young Adults

Parameter/subgroup	Sleep		Pain		Anxiety		Depression		Fatigue		Physical function impairment	
	OR^a^	*P* value	OR^a^	*P* value	OR^a^	*P* value	OR^a^	*P* value	OR^a^	*P* value	OR^a^	*P* value
Female sex (reference group, male)	NA	NA	NA	NA	1.65 (1.27-2.14)	<.001	1.50 (1.09-2.06)	.01	NA	NA	NA	NA
Race (reference group, White)												
Additional groups^b^	NA		1.65 (1.12- 2.45)	<.01	1.41 (1.01-1.99)	.05	1.52 (1.02- 2.26)	.04	NA	NA	1.41 (1.09- 1.82)	.12
Area code (reference group, urban)												
Micropolitan	NA	NA	1.62 (1.10- 2.38)	0.01	1.56 (1.14-2.15)	.006	1.78 (1.21- 2.60)	.003	NA	NA	1.99 (1.32- 2.97)	<.001
Rural	NA	NA	1.14 (0.79- 1.65)	0.47	0.77 (0.55-1.07)	.12	1.03 (0.70- 1.52)	0.88	NA	NA	1.25 (0.84- 1.88)	.27
Marital status (reference group, married or partnered)												
Divorced or separated	1.98 (1.18- 3.31)	.009	NA	NA	NA	NA	NA	NA	2.33 (1.43- 3.81)	0.003	NA	NA
Single	1.36 (1.05- 1.76)	.02	NA	NA	NA	NA	NA	NA	1.27 (0.99- 1.64)	0.12	NA	NA
Insurance payer (reference group, non-government)												
Government	1.94 (1.48- 2.55)	<.001	1.93 (1.38- 2.70)	<.001	1.57 (1.18- 2.08)	.002	1.49 (1.06- 2.09)	.02	1.57 (1.18- 2.08)	.002	1.37 (0.94- 1.99)	.09
Employment (reference group, employed)												
Disabled	NA	NA	3.50 (2.06- 5.97)	<.001	2.26 (1.36-3.77)	.002	2.80 (1.58- 4.94)	<.001	2.26 (1.40-3.65)	.002	2.30 (1.22- 4.35)	.01
Not employed, student, or military	NA	NA	1.74 (1.25- 2.42)	<.001	1.34 (1.01-1.77)	.04	1.60 (1.15-2.23)	.006	1.34 (1.01- 1.76)	.04	1.67 (1.16- 2.38)	.005
Primary malignant neoplasm(reference group, solid)												
Hematologic	NA	NA	NA	NA	1.49 (0.93- 2.41)	.13	NA	NA	1.49 (0.93- 2.41)	.13	NA	NA

Abbreviations: NA, not applicable; OR, odds ratio.

^a^ An OR greater than 1 suggests a greater likelihood of a severe symptom compared with the reference group, while an OR less than 1 suggests a lower likelihood of a severe symptom compared with the reference group. Statistical significance was defined as *P* < .008 per Bonferroni correction (ie, .05 divided by 6 for the 6 symptom models). Squares marked NA indicate that that risk factor was not included in multivariable analysis for that specific SPPADE symptom because it was not significant on univariate analysis.

^b^ Additional racial groups included African American, Asian or Pacific Islander, and other or unknown.

## Discussion

This cross-sectional study is one of the largest to assess cancer symptom burden in AYAs using patient-reported outcomes. AYAs experienced high rates of moderate and severe anxiety and depression. This is of importance considering alarming increases in rates of death by suicide among AYA patients in recent years.^5^ Limitations of this secondary analysis from a large pragmatic trial include inability to control for time since diagnosis and prior treatment history. Further, patients treated at Mayo Clinic may not be representative of the general population. However, our findings provide evidence that interventions focused on mental health are needed for AYAs with cancer and highlight characteristics of AYAs who may benefit most from symptom-focused interventions.

## Notes

Supplement 1.
eMethods.

Supplement 2.
Data Sharing Statement
