# Supplementary material for: Symptom Burden in Adolescents and Young Adults With Cancer
Source: JAMA Netw Open. 2025 Aug 18;8(8):e2527421. doi: 10.1001/jamanetworkopen.2025.27421 (PMC12362219; doi:10.1001/jamanetworkopen.2025.27421)
Supplement: Supplement 1. — eMethods. [file jamanetwopen-e2527421-s001.pdf]

Supplemental Online Content

Storandt MH, Jin Z, Ruddy KJ, et al. Symptom burden in adolescents and young adults with cancer. *JAMA Netw Open.* 2025;8(8):e2527421.
doi:10.1001/jamanetworkopen.2025.27421

This supplemental material has been provided by the authors to give readers additional information about their work.

© 2025 Storandt MH et al. *JAMA Network Open*.

eMethods

Participants

The E2C2 trial enrolled adult patients receiving care for a solid malignancy through the Department of Medical Oncology at Mayo Clinic Rochester, or a solid or hematologic malignancy within the Mayo Clinic Health System (MCHS) in Minnesota or Wisconsin. The trial utilized inclusive enrollment, with all eligible adult patients enrolled in the trial. Eligible patients included those who were receiving treatment for a cancer, being surveilled for cancer recurrence, and/or receiving survivorship care. In this post hoc analysis, we assessed all patients who had completed at least one SPPADE questionnaire, specifically assessing the first SPPADE questionnaire completed by each patient. Patients were stratified by age of completion of their first questionnaire, with 18-39 years defined as AYA.

Measures

Patient demographic information, including sex, race, ethnicity, marital status, employment, and insurance coverage was extracted from the EHR. Rural Urban Commuting Area (RUCA) codes were used to classify degree of rurality using patient zip codes. In the present analysis, all individuals of non-white race were grouped, given a low number of patients from non-white racial groups included in this study, to allow for statistical analysis. The indication for inclusion of race in our analyses was to assess disparities in patient symptom burden between a white population and those of a minority race.

SPPADE symptoms were measured using an 11-point NRS as noted. Symptom severity was classified as none to mild (0-3), moderate (4-6), or severe (7-10), as previously validated (Jeon et al., *J Pain Symptom Manage*. 2009, Woo et al., *Ann Palliat Med*. 2015).

Statistical analysis

Univariate analysis for each SPPADE symptom was performed to examine demographic and tumor characteristics associated with having a severe symptom among AYAs. Patient demographic characteristics included age, sex, race, ethnicity, population density of home community, marital status, insurance payer, employment status, and educational status. Tumor characteristics assessed included

© 2025 Storandt MH et al. *JAMA Network Open*.

primary tumor location and metastatic disease status. Logistic regression was used for each demographic or tumor characteristic, providing an odds ratio and *p*-value comparing each subgroup within each characteristic against a reference group, typically the one with the largest frequency. We then completed a multivariable logistic regression analysis. Backwards stepwise regression was used for model selection, providing significant predictors for each SPPADE symptom. Odds ratios are reported to reflect the degree of association between demographic and clinical factors, and each of the SPPADE symptoms.

© 2025 Storandt MH et al. *JAMA Network Open*.
