# Supplementary material for: Symptom Burden in Adolescents and Young Adults With Cancer
Source: JAMA Netw Open. 2025 Aug 18;8(8):e2527421. doi: 10.1001/jamanetworkopen.2025.27421 (PMC12362219; doi:10.1001/jamanetworkopen.2025.27421)
Supplement: Supplement 2. — Data Sharing Statement [file jamanetwopen-e2527421-s002.pdf]

Data Sharing Statement

Storandt. Symptom Burden in Adolescents and Young Adults With Cancer. *JAMA Netw Open*. Published August 18, 2025. doi:10.1001/jamanetworkopen.2025.27421

Data
Data available: Yes
Data types: Deidentified participant data, Data dictionary
How to access data: With publication, deidentified participant data, as well as the data dictionary and study protocol will be made available upon request to cheville.andrea@mayo.edu and through the Harvard Dataverse.
When available: With publication

Supporting Documents
Document types: None

Additional Information
Who can access the data: With publication, deidentified participant data, as well as the data dictionary and study protocol will be made available upon request to cheville.andrea@mayo.edu and through the Harvard Dataverse.
Types of analyses: With publication, deidentified participant data, as well as the data dictionary and study protocol will be made available upon request to cheville.andrea@mayo.edu and through the Harvard Dataverse.
Mechanisms of data availability: With publication, deidentified participant data, as well as the data dictionary and study protocol will be made available upon request to cheville.andrea@mayo.edu and through the Harvard Dataverse.
